# Infrared Spectroscopy on Equilibrated High-Density Amorphous Ice

**DOI:** 10.1021/acs.jpclett.2c02074

**Published:** 2022-08-18

**Authors:** Aigerim Karina, Tobias Eklund, Christina M. Tonauer, Hailong Li, Thomas Loerting, Katrin Amann-Winkel

**Affiliations:** † Department of Physics, AlbaNova University Center, Stockholm University, SE-10691 Stockholm, Sweden; ‡ Institute of Physics, Johannes Gutenberg University Mainz, 55128 Mainz, Germany; § Institute of Physical Chemistry, University of Innsbruck, A-6020 Innsbruck, Austria; ∥ Max-Planck-Institute for Polymer Research, 55128 Mainz, Germany

## Abstract

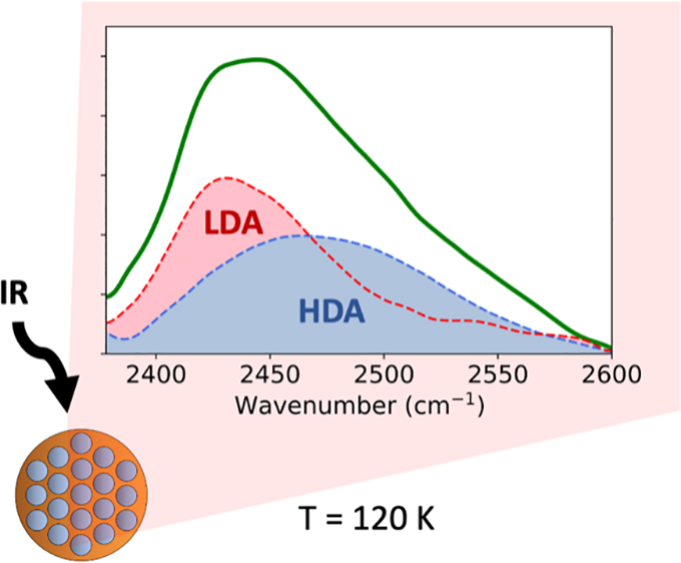

High-density (HDA) and low-density amorphous ices (LDA) are believed to be counterparts of the high- and low-density liquid phases of water, respectively. In order to better understand how the vibrational modes change during the transition between the two solid states, we present infrared spectroscopy measurements, following the change of the decoupled OD-stretch (*v*_*OD*_) (∼2460 cm^–1^) and OH-combinational mode (*v*_*OH*_ + *v*_2_, *v*_*OH*_ + 2*v*_*R*_) (∼5000 cm^–1^). We observe a redshift from HDA to LDA, accompanied with a drastic decrease of the bandwidth. The hydrogen bonds are stronger in LDA, which is caused by a change in the coordination number and number of water molecules interstitial between the first and second hydration shell. The unusually broad uncoupled OD band also clearly distinguishes HDA from other crystalline high-pressure phases, while the shape and position of the in situ prepared LDA are comparable to those of vapor-deposited amorphous ice.

Water molecules in ice can be either arranged in a crystalline lattice or appear disordered in an amorphous solid. Such amorphous ices can be found naturally in outer space^1,2^ and in very cold mesospheric clouds in Earth’s atmosphere^3^ but also have found application in cryo-electron microscopy.^4^ Water’s ability to form at least two different forms of amorphous ice^5^ is connected to our fundamental understanding of water’s phase diagram and represents the most famous case of polyamorphism in a one-component system.^6^ This is, when hexagonal ice Ih is compressed to 1.6 GPa at 77 K, it forms high-density amorphous ice (HDA),^7^ which can transform to low-density amorphous ice (LDA) when decompressed at around 140 K^8^ or heated at ambient pressure.^9^ This polyamorphic transition is suggested to be linked to a liquid–liquid transition (LLT) at 140 K and above^6,8,10,11^ between high- (HDL) and low-density liquid (LDL). This scenario observed in computer simulations of different water models^10,11^ was accessed in slow decompression experiments at 140 K just below the crystallization line^8^ and became recently experimentally accessible also at higher temperatures by ultrafast laser heating, allowing to probe the LLT by X-ray scattering at slightly elevated pressure and temperatures where usually crystallization occurs.^12^ At ambient pressure and low temperatures, the metastable amorphous states and their conversion have been intensively studied using different experimental methods such as X-ray^8,13,14^ and neutron diffraction,^15,16^ calorimetry,^8,17,18^ broadband dielectric relaxation,^17,19^ and deuteron and ^17^O NMR^20,21^ spectroscopy. The vibrational spectrum of amorphous ices was previously accessed using Raman spectroscopy^22,23^ and incoherent inelastic neutron scattering.^24−26^ Additionally, infrared spectroscopy allows studying amorphous ices at the molecular level by measuring vibrational states of hydrogen bonds.^27,28^ This is of particular interest for a comparison with astrophysical data.^1,29^ The low-density amorphous state of water grown by vapor deposition is studied intensively using infrared spectroscopy.^30−34^ However, no IR data of the high-density forms obtained by pressure induced amorphization have so far been reported. Water unlike other liquids absorbs strongly in the mid-IR region. This property limits the thickness of water samples in the transmission geometry down to a few micrometers. We have overcome these experimental challenges by preparing HDA ice samples as free-standing 50–80 μm thick layers that can be measured at cryogenic temperatures in vacuum, without protecting windows.

Here, we present Fourier transform mid-infrared spectrometry (FTIR) spectroscopy measurements in transmission geometry and measurements in diffuse reflection geometry using a Fourier transform near-infrared spectrometer (FTNIR).^35^ For this, we prepared equilibrated HDA (eHDA)^36^ through a well-established thermal annealing pathway at elevated pressures.^12,17,37^ Samples have been prepared in a piston-cylinder setup as bulk samples for the measurements in diffuse reflection geometry, while for the transmission measurements, a 100 μm thick copper grid is used to support the ice film (Figure 1). X-ray measurements confirm that eHDA is formed inside the grid-holes;^12^ the measured structure factor *S*(*Q*) is identical to the one measured from bulk samples. From X-ray studies on bulk and grid samples,^38−40^ it is well-known that HDA upon warming transforms to the low-density state; the development of *S*(*Q*) is identical for both sample types. The process is exothermic^16^ and involves a volume change^8^ of 20%; for the grid samples, the expansion can take place perpendicular to the grid, as no windows restrain the motion. Here, we now follow this transition using FTIR.

**Figure 1:**
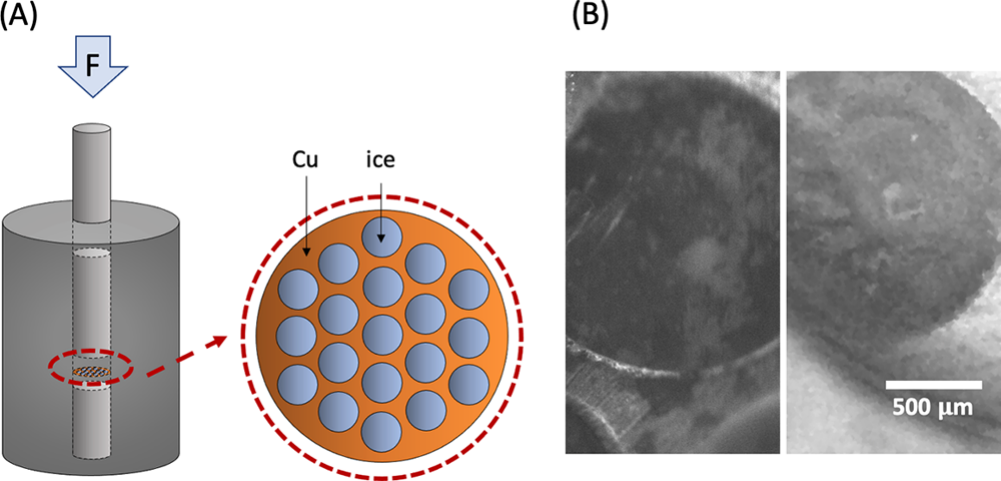
(A) High-pressure cell setup for the eHDA sample preparation. (B) Pictures of ice in the copper grid-holes made by an Infinity K2/DistaMax Long Distance microscope.

Figure 2 shows the recorded FTIR spectrum (blue) of the eHDA sample at 80 K. Absorbance *A* at a certain wavenumber ν is, according to Beer’s Law, a logarithmic ratio of initial power of radiation *I*_0_ to the radiant power transmitted *I* through the sample
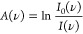
1

**Figure 2:**
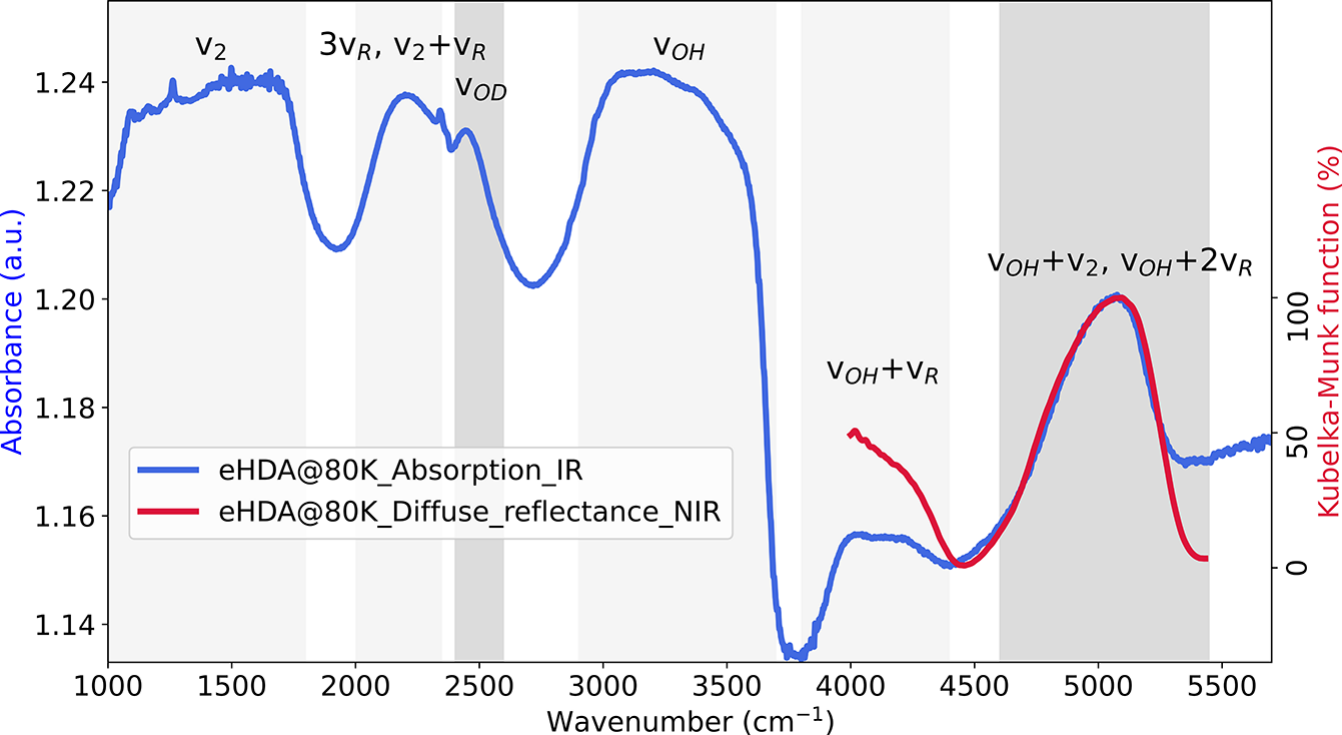
Uncorrected FTIR spectra of eHDA in a copper grid (blue) in the range of 1000–5700 cm^–1^ at 80 K. The OH-combinational region of the thin eHDA sample is compared to the diffuse reflectance measurements of a thick, powdered eHDA sample, depicted as a Kubelka–Munk or remission function spectrum (red).

Due to the still relatively thick sample, the OH stretch region around 3200 cm^–1^ is saturated. We used an isotopically diluted solution^41^ of 1 wt.% HOD in H_2_O to look at decoupled OD-stretching bands in the range of 2400–2600 cm^–1^. Due to the small amount of deuterium, the OD mode appears as a small peak on the high-frequency wing of the combinational mode of the HOH bending mode (*v*_2_) and water libration (*v*_*R*_). Background subtraction is discussed in Figure S2. The combinational modes *v*_*OH*_ + *v*_2_ and *v*_*OH*_ + 2*v*_*R*_, at around 5000 cm^–1^ are much weaker than the OH stretch mode, so they are not saturated in spite of the thickness of the sample. This provides us information about the OH-stretch mode. This region has been additionally studied by using a diffuse reflectance geometry in an FTNIR spectrometer (red line). Reflectance was converted to Kubelka–Munk or remission function (),^42^ according to eq 2
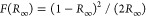
2where *R*_*∞*_ denotes the measured reflectance of a sample thick enough that transmission is negligible. Both spectra are in very good agreement, even though taken with different experimental methods. The band position is found to be at 5048 cm^–1^ for the absorbance measurements and at 5082 cm^–1^ for the diffuse reflectance data. The broad line shape of the decoupled OD-stretch band of eHDA, with a full width at half-maximum (fwhm) of 118 cm^–1^ is similar—in terms of width and shape—to liquid water.^31^ The HDA band is clearly distinguished from other high-pressure ice phases (see Figure S1). Even though its center position is similar to the IR spectra of ice V and VI, the eHDA spectrum does not contain any subpeaks, as the crystalline ices. Also, the fwhm is significantly broader compared to the hydrogen disordered crystalline ices V and VI, a feature which had been reported to indicate a broad range of OH frequencies and bond lengths (Figure S1). For eHDA, this appears to be even more pronounced, consistent with both oxygen and hydrogen disorder.

Subsequently, we recorded spectra while heating eHDA from 80 to 160 K in steps of 5–10 K, as shown in Figure 3. All spectra are collected after quenching back to 80 K. For the OD-stretch FTIR spectra of eHDA at different temperatures, we first subtracted a linear baseline and normalized the spectra to the peak maximum (Figure S2), while for the OH-combinational (*v*_*OH*_ + *v*_2_, *v*_*OH*_ + 2*v*_*R*_) mode, we subtracted individual linear baselines and normalized the spectra to the peak maximum (for details, see SI). In Figures 3A,B, we can visually observe that curves obtained after heating to 90–115 K (blue curves) have similar broadness and peak positions, which show that eHDA can be kept stable at these temperatures.

**Figure 3:**
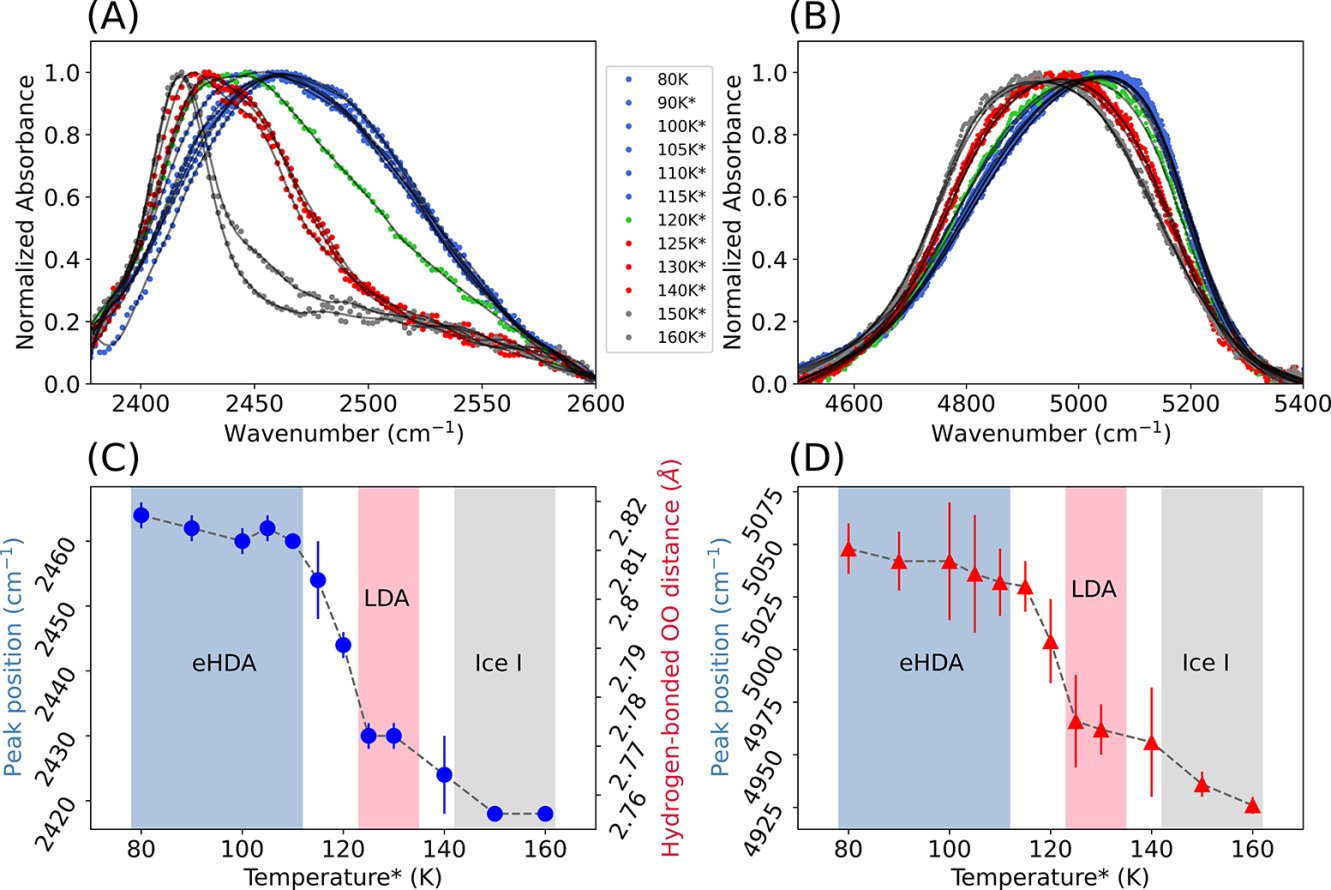
(A) Baseline-corrected FTIR spectra of the OD-stretch mode and (B) OH-combinational mode of the eHDA sample normalized to the peak maximum. The circles represent raw data (colorful dots), and the gray solid lines are results of a Savitzky-Golay filter application. (C,D) Peak positions of OD-stretch and OH-combinational mode signals as a function of temperature. (C) Additionally, the average distance of the hydrogen-bonded pair of oxygen atoms is presented on the right axis. *Measurements are taken at 80 K after heating to corresponding temperatures and annealing for 10 min.

Above 120 K, we observe a shift of the maximum toward lower wavenumbers for the OD-stretch and OH-combination bands, respectively, visible also from the peak positions in Figure 3C,D. For the OD-stretch band, the peak shift is accompanied by a narrowing of the spectrum, while the low-frequency wing at around 2400 cm^–1^ remains. The sample remains metastable in the low-density state at 125–140 K. This temperature range is consistent with recent X-ray data taken on similarly prepared eHDA samples.^38,39^ At the crystallization temperature, the peak maximum is shifted further toward lower wavenumbers, an indication of stronger hydrogen bonds. IR spectroscopy probes the local environment; from the measured O–H stretching frequencies, the hydrogen bond length in crystals and minerals can be calculated.^43,44^ The vibrational frequency of an uncoupled O–D bond is strongly correlated to the distance of the nearest neighbor hydrogen-bonded oxygen atoms. The correlation is well-established empirically^45,46^ and theoretically;^47,48^ therefore, the vibrational frequency *v*_*OD*_ can be converted to the average O–H···O distance *R* by the following equation^49^
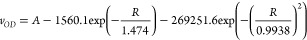
3where *A* is the O–D frequency of the isolated HDO molecule (2782.1 cm^–1^). We calculated the average hydrogen-bonded O–O distances for eHDA (2.816 Å) and LDA (2.771 Å). The calculated values are consistent with values obtained from Raman measurements.^23,49,50^ The O–O distance as a function of the temperature is presented in Figure 3C on the right axis. Please note that the redshift on the polyamorphic transition goes together with a decrease in O–O distance. This counterintuitive observation of shorter, stronger H-bonds in the less dense material observed at lower vibrational energies can be explained by the density-distance paradox.^13,23^ In essence, the O–O distance is longer in HDA because a molecule moves from the second coordination shell to the space interstitial between the first and second shell, where the first shell needs to provide some more space to accommodate the additional neighboring molecule. The same trend was observed when calculating the O–O distance from X-ray measurements^35^ on protonated eHDA samples, where the distance to the first nearest oxygen neighbor was extracted from the first maximum in the pair distribution function (PDF) to increase from *r* = 2.750 Å for LDA to 2.780 Å for HDA. The ratio of the first and second maxima in the PDF instead provides information for the tetrahedrality and is for LDA found to be very close to 1.633, the tetrahedral O–O–O angle. The coordination number can be calculated by integrating the PDF, and X-ray^35^ and neutron scattering^15^ data both show a change in coordination number of 4 + 1 in HDA to 4 in LDA. This causes the redshift in the FTIR data and a sharpening of the band to an fwhm of 33 cm^–1^. How do the vibrational modes of the so-derived LDA and crystalline ice compare to vapor-deposited amorphous solid water (ASW) and other crystalline ices? A comparison of the different states and references (30), (33), (51), and (52) of ice Ih and amorphous solid water (ASW) is given in Table 1. Most importantly, the peak position and fwhm of the derived LDA are identical to ASW, as also visible in Figure S4. This is consistent with X-ray and neutron data, demonstrating that ASW is a structural analogue to LDA.^16,33^ This finding is in contrast to work by Kolesnikov et al.,^24^ who observed considerable differences between the vapor deposits and the LDA obtained from HDA. The difference in their study might actually be due to the microporous nature of ASW, resulting in many molecules that are not tetrahedrally coordinated, as compared to the compact nature and perfect tetrahedral coordination in LDA. That is to say that it needs to be clarified how porous or how compact the vapor deposit actually is—only well-annealed ASW samples (e.g., at 120 K) are similar to compact LDA.^53^

**Table 1:** OD-Stretch Modes Measured at around 80 K by FTIR and OH-Combination Modes Measured by FTIR and Diffuse Reflectance NIR in Comparison with the Literature

	*v*_*OD*_ peak position (cm^–1^)	*v*_*OD*_ fwhm (cm^–1^)	*v*_2_ + *v*_*OH*_, *v*_*R*_ + *v*_*OH*_ peak position (cm^–1^)	*v*_2_ + *v*_*OH*_, *v*_*R*_ + *v*_*OH*_ fwhm (cm^–1^)
Ice-Ih	2420 (Bergren et al., 1978); 2422 (±2) (this work)	20 (Bergren et al., 1978); 35 (±2) (this work)	4983 (Grundy et al., 1998); 4971 (Tonauer et al., 2021)	600 (±40) (Grundy et al., 1998); 566 (Tonauer et al., 2021)
Ice-Isd (from eHDA)	2418 (±2)	33 (±2)	4925 (±10)	418 (±10)
Ice-Isd (from ASW)	2418 (±2) (Li et al., 2021)	32 (±2) (Li et al., 2021)	-	-
ASW	2439 (Bergren et al., 1978)	70 (Bergren et al., 1978)	4998 (Mastrapa et al., 2008)	380 (±10) Mastrapa et al., 2008
LDA (Absorption)	2432 (±2)	73 (±2)	4966 (±10)	427 (±10)
LDA (Diff. reflectance)	-	-	4997 (±10)	513 (±10)
eHDA (Absorption)	2464 (±2)	118 (±2)	5048 (±10)	418 (±10)
eHDA (Diff. reflectance)	-	-	5082 (±10)	496 (±10)

We further discuss differently prepared crystalline ices, namely cubic ice crystallized here from LDA at 160 K and hexagonal ice obtained by directly freezing water in such a copper grid, freezing water between CaF_2_ windows, and from crystallizing ASW at 160 K.^33^ Both hexagonal ice samples, hence prepared directly from freezing liquid water, have peak maxima at 2422 cm^–1^ (see also Figure S5). The peak maximum of cubic ice obtained after the transition eHDA → LDA and annealing ASW is located at 2418 cm^–1^. We relate this to the so-called stacking disordered ice (Isd) formation, which has a slightly different OD-stretch vibrational frequency than hexagonal ice.^54^ Formation of Isd from heating LDA or ASW was already reported in several works^18,39,53−55^ and is consistent with recent X-ray data taken at such grid samples.^58^ We here find a O–O distance for ice Isd of *R* = 2.755 Å. This is, we observe eHDA to be stable in the range 80–115 K as well sa LDA in the range 125–140 K and observe crystallization at 150 K.

An interesting observation in this series of measurements is the signal of the sample annealed to 120 K. The spectrum at 120 K represents a mixture of eHDA as the initial state and LDA as the final state of the transformation. We show a linear combination (black dashed line) of eHDA at 80 K (blue) and LDA (quenched from 130 K) (red) of different proportions. The results are compared with the sample annealed to 120 K, and the best matches are presented in Figure 4A,B. Analyzing the OD-stretch band, we find 60% LDA and 40% eHDA at 120 K, while 40% LDA and 60% eHDA is found comparing the OH-combination band at the same temperature. This difference could simply be related to the overlapping contributions in the combinational band but still represents an approximate 50% coexistence of the two states at this temperature.

**Figure 4:**
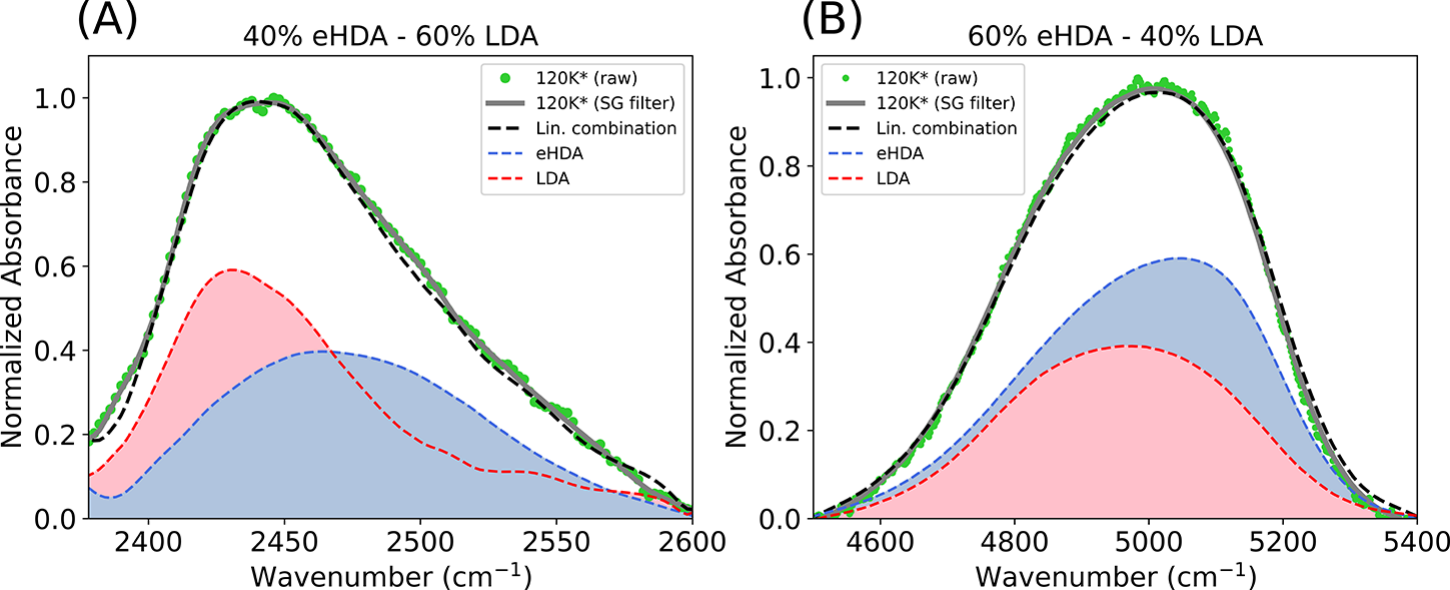
(A) Comparison of the FTIR spectrum of the OD-stretch mode after annealing eHDA to 120 K and the linear combination of eHDA and LDA signals. (B) Comparison of the FTIR spectrum of the OH-combinational mode after annealing eHDA to 120 K and the linear combination of eHDA and LDA signals. *Measurements are taken at 80 K after heating to corresponding temperatures.

In summary, we have demonstrated by IR measurements how the strength of the hydrogen bonds increases within the course of the transformation from eHDA to LDA and ice Isd. This becomes visible through the redshift of the decoupled OD-stretch peak and a decrease in the fwhm of the spectra from 118 cm^–1^ for eHDA to 73 cm^–1^ for LDA, while the low-frequency wing remains at a similar position. Through the empirical link between vibrational frequency and O–O distance, we showed that a shortening of the O–O distance is observed at the polyamorphic transition, consistent with X-ray data.^35^ Comparing the decoupled OD-modes of hexagonal ice and liquid water,^31,59^ the here observed spectral features of the decoupled OD-stretch band of HDA are more similar to warm liquid water^60^ rather than other high-pressure ice phases (Figure S1), indicating more disorder. For liquid water, a low-frequency band shift accompanied by an increase in intensity and narrowing of the OH stretch (∼3200 cm^–1^) mode was observed in experiments and simulations when water is supercooled.^59−61^ Likewise, also the X-ray PDF of HDA is more similar to water at 365.9 K, while the PDF of LDA is more similar to supercooled water,^35^ interpreted with an increase of tetrahedrality.^6^ This is, our IR data are consistent with the hypothesis that warm water is more of a high-density structure, while fluctuations of low-density structures appear at lower temperatures.^6^ Shape and position for *v*_*OD*_ of LDA are found to be identical to a well-annealed vapor-deposited ASW after the collapse of micropores. Highly microporous ASW samples deposited at <100 K show different spectra due to the high surface area and a large fraction of molecules that are not fully coordinated, with much more dangling OH bonds. Phase coexistence of eHDA and LDA is observed at around 120 K, where the intermediate spectrum can be reconstructed by a linear combination of the two pure states, here demonstrated for the OD-stretch band as well as the combinational mode around 5000 cm^–1^. This coexistence has already been reported in optical studies by Mishima^62^ and later demonstrated in decompression experiments through neutron^63^ and X-ray diffraction.^8^ Here, we present that IR spectra from different intermediate temperatures can be fitted by a linear combination of starting and final state. This adds another important feature demonstrating the first-order-like nature of the HDA → LDA transition, while recent X-ray experiments have also confirmed their diffusive nature.^14^

## Experimental Methods

Samples were prepared in a piston-cylinder setup, as powder samples for the diffuse reflectance measurements, while for the measurements in transmission geometry, a 100 μm thick copper grid is used to support the ice film (Figure 1). The copper grid samples were prepared at Stockholm University. The grid with holes of 1.5 mm in diameter is dipped in ultrapure water, which is subsequently frozen to hexagonal ice before being assembled to the piston cylinder. The bulk eHDA sample was prepared at University of Innsbruck, using 600 μL of ultrapure water pipetted to an indium container following the same T-P pathway. Powder made from the bulk sample with 1 mm thickness was used for near-infrared spectroscopy measurements.

The absorbance infrared measurements were obtained with an FTIR spectrometer (Frontier, PerkinElmer) in a range of 6000–1000 cm^–1^ with a resolution of 2 cm^–1^. Each spectrum was collected for 1 min and six scans. The sample had been mounted in a temperature-controlled liquid nitrogen cryostat (VPF 100, Janis) and measured through IR-polished CaF_2_ optical windows. The reported temperatures are not measured directly at the sample itself but rather at the cryostat head and are therefore assumed to have a slight offset. For the measurements in diffuse reflectance geometry, a Büchi NIR Flex N-500 benchtop Fourier transform near-infrared spectrometer (10 000–4000 cm^–1^) was utilized. At least three independently prepared samples of the two polyamorphs were analyzed, adding up to at least 20 cumulative spectra per ice polyamorph. One cumulative spectrum was recorded within 16 s and represents a sum of 32 single spectra at a resolution of 8 cm^–1^.

## Supporting Information Available

Comparison of eHDA’s IR spectra with high-pressure ices V and VI, details of baseline subtraction, comparison of differently prepared (eHDA, LDA, porous and compact ASW) and measured (transmission, diffuse reflection) amorphous ices and crystalline ices derived from them, detailed slow conversion of eHDA to LDA at 90, 105, and 115 K (PDF)
