# Supplementary material for: Infrared Spectroscopy on Equilibrated High-Density Amorphous Ice
Source: J Phys Chem Lett. 2022 Aug 18;13(34):7965–71. doi: 10.1021/acs.jpclett.2c02074 (PMC9442797; doi:10.1021/acs.jpclett.2c02074)
Supplement: Supplementary file 1 — jz2c02074_si_001.pdf [file jz2c02074_si_001.pdf]

Supporting Information

Infrared Spectroscopy on Equilibrated High-Density Amorphous Ice

Aigerim Karina[1], Tobias Eklund[1,2], Christina M. Tonauer[3], Hailong Li[4], Thomas Loerting[3], Katrin Amann-Winkel[1,2,4,*]

1. Department of Physics, Stockholm University, SE-10691 Stockholm, Sweden.

2. Institute of Physics, Johannes Gutenberg University Mainz, 55128 Mainz, Germany

3. Institute of Physical Chemistry, University of Innsbruck, A-6020 Innsbruck, Austria

4. Max-Planck-Institute for Polymer Research, 55128 Mainz, Germany

* Corresponding author, email address: amannk@mpip-mainz.mpg.de

In the main manuscript, we present infrared spectroscopy measurements, following the transition between equilibrated high- and low-density amorphous ice (eHDA, LDA). We used an isotopically diluted solution of 1% HOD in H_2O to look at decoupled OD-stretching bands in the range of 2400-2600 cm^{-1}, and additionally investigated the combinational modes ($\nu_{OH}+\nu_2$, $\nu_{OH}+2\nu_R$) at around 5000 cm^{-1}. A new sample preparation method allowed FTIR measurements in transmission geometry, for details see main manuscript.

First, the uncoupled OD-stretch mode of equilibrated high-density amorphous ice (eHDA) is compared with the modes of two **high-pressure crystalline ices**, ice V and ice VI (Fig. S1A). Both crystalline ices exhibit broad full width at half maximum (FWHM), which is due to hydrogen disorder in both ice phases. Moreover, significant shoulders indicate the presence of well-defined order in ice V, although not very prominent in ice VI. In literature a wide range of tetrahedral O-O-O angle in ice VI is assigned to contributes to the line broadening[1]. However, the FWHM of eHDA is with 118 cm^{-1} extraordinary broad and results from disorder

of oxygen (and hydrogen) atoms in amorphous ice. In Figure S1B we compare the combinational mode of eHDA measured in the diffuse reflectance mode with ice V and VI measured at the same setup[2]. The FWHM of eHDA is broader than the FWHM of crystalline ices. The peak position of eHDA is shifted towards higher wavenumbers.

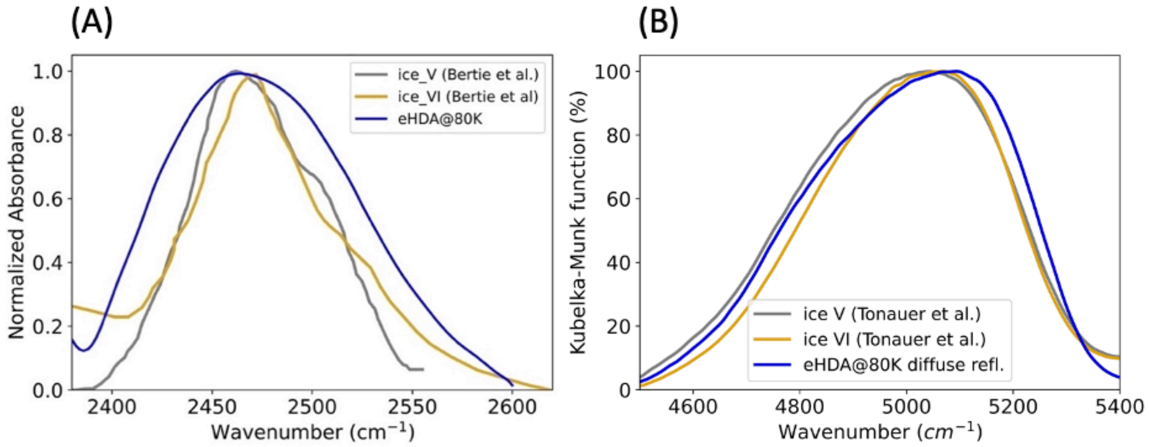

Figure S1. Comparison of the uncoupled OD-stretch (A) and the combinational modes (B) of eHDA and high-pressure crystalline ices from *Bertie et al*[3,4] and *Tonauer et al.*[2]

Data treatment: The OD-stretch mode overlaps with the strong bending and libration combination mode $3\nu_R$, $\nu_2+\nu_R$ in the range of 2000 – 2600 cm^{-1}. However, the main peak is well separated from the OD mode, which is sitting at the high-frequency wing. In order to analyze the OD spectra, we therefore subtracted a linear baseline in a range from 2262 cm^{-1} to 2600 cm^{-1} shown in Fig. S2A. In the resulting spectra (Fig. S2B) we observe a decrease of the intensity due to some loss of the sample in vacuum. This is mainly observed during the heating-cooling cycle between the annealing temperatures and 80 K, where all spectra have been recorded. It can be seen as well that the intensity of CO_2 becomes negative at higher temperatures, resulting from the sample loss. (More detailed, the software of FTIR spectrometer subtracts the background with initial larger amount of CO_2 automatically after each measurement.) Since the HDA spectra obtained after heating to 90 K – 115 K (blue curves) have similar broadness and peak positions despite getting lower in intensity, we relate

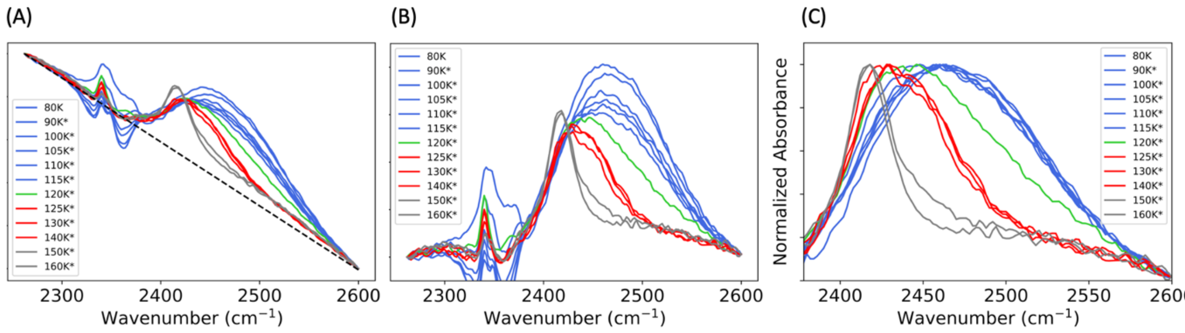

Figure S2. A) The linear baseline subtracted from the OD spectra and B) the result of the subtraction. C) The resulting spectra normalized to the height. *Measurements are taken at 80 K after heating to corresponding temperatures and annealing for 10min.

the material loss mainly to the heating-cooling cycle. The transition from HDA to LDA is accompanied by a density change of 20%, also this effect might contribute to material loss at higher temperatures. We visually observe the sample to be still intact and not all samples show a similar amount of sample loss, as this also depends on the thermal contact of the ice inside the grid-hole. Nevertheless, the sample can expand in the two directions perpendicular to the grid, as no window material protects the sample. We therefore normalized the spectra to the height (Fig. S2C) for better following the change of the line shape as well as FWHM during the transitions.

For most of the combinational mode curves no baseline correction was necessary, as most spectra were flat, except 80 K, 90 K*, 120 K*, and 130 K* (Fig. S3). (*Measurements are taken

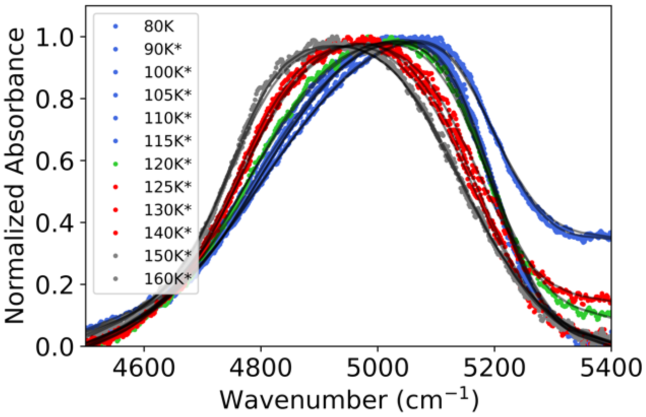

Figure S3. Uncorrected combinational mode ($\nu_{OH}+\nu_2$, $\nu_{OH}+2\nu_R$) spectra.

at 80 K after heating to corresponding temperatures and annealing for 10min.) The last curves seem to have a linear increase towards higher wavenumbers. We relate it to light scattering from the copper grid, where the latter expands and shrinks at higher and lower temperatures, respectively, when we heat or quench the sample.

Comparison to vapor deposited ice (ASW):

In our previous work[5] we prepared vapor-deposited amorphous ice (ASW) which was found to be structural analog of low-density amorphous ice (LDA) derived from heating eHDA according to X-ray studies. We here compare the OD-stretch mode of eHDA, LDA derived from eHDA with ASW (Fig. S4). Unannealed ASW is known to be a porous material and due to that shifted towards higher wavenumber. Therefore, we compared our sample with both porous ASW (p-ASW) and LDA derived from annealing p-ASW to compact ASW (c-ASW). Both, differently prepared LDAs have similar FWHM as well as similar peak positions. Ice I_{sd} obtained from eHDA>LDA>I_{sd} and p-ASW>c-ASW>I_{sd} transitions are found to have identical vibrational spectra.

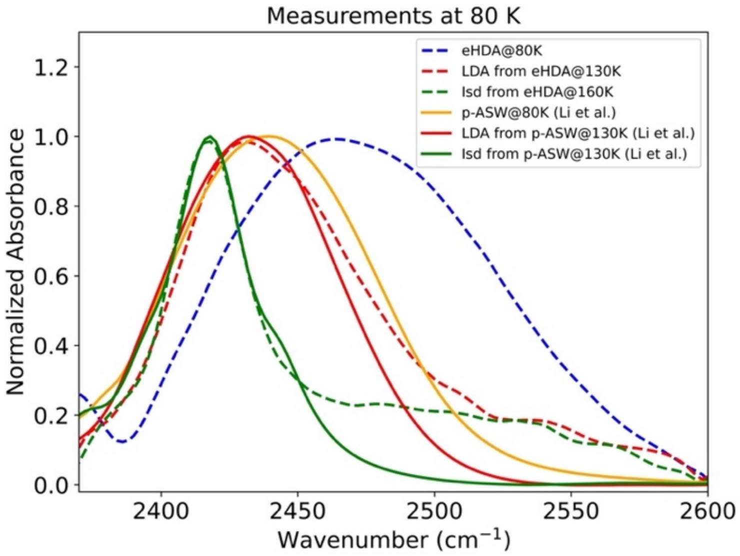

Figure S4. Comparison of OD stretch mode of eHDA at 80 K then annealed to 130 K, 160 K with p-ASW, c-ASW and stacking disorder ice (Isd) from our previous work[5].

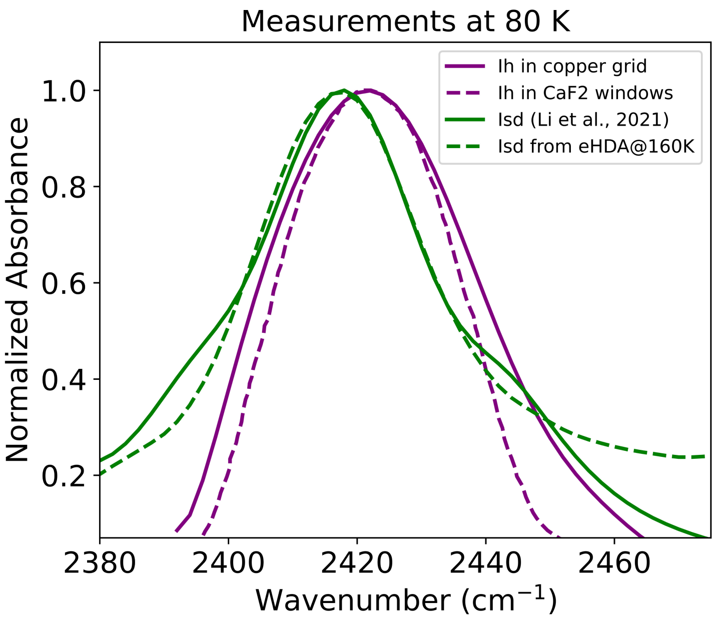

Figure S5. OD stretch mode comparison of ices obtained by annealing eHDA and ASW[5] with ices prepared directly by freezing water in LN_2.

An additional comparison to cubic ice (precisely to be called stacking disordered ice Isd, as not fully cubic) derived from heating vapor deposited ice to 160 K, is shown in Figure S5. Interestingly the spectrum of the so derived crystalline ice (green solid line) is identical to the spectrum of crystalline ice after heating eHDA to 160 K (green dashed line). Both are clearly distinct from hexagonal ice prepared separately. All the spectra in Fig. S4 and S5 are measured at 80 K.

Comparison of combinational mode ($\nu_{OH}+\nu_2$, $\nu_{OH}+2\nu_R$) of ASW with LDA derived from eHDA is technically limited. Vapor deposited ASW samples are usually too thin for measuring relatively weaker mode at ~5000 cm^{-1}. We here compare a 5 µm thick ASW sample we had grown for 4 hours[5]. The comparison is presented in Fig. S6A, where we can see that the peaks positions of LDA and ASW are identical.

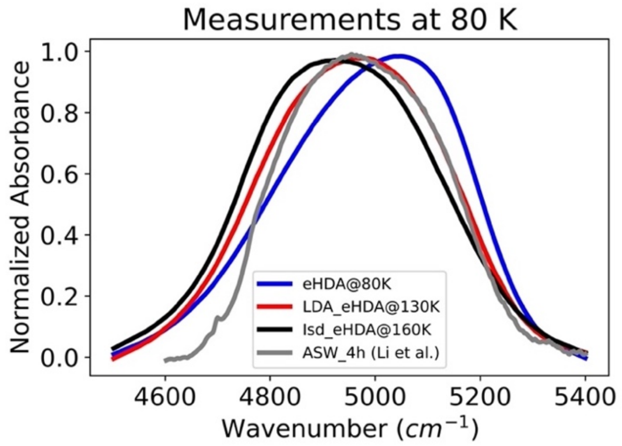

Figure S6A: Comparison of combination mode of eHDA sample at 80 K and after annealing to 130 K, 160 K and ASW grown by vapor deposition[5]. All spectra were measured at 80 K.

Two different experimental FTIR techniques:

In the main manuscript we present Fourier-transform mid-infrared spectrometry (FTIR) spectroscopy measurements in transmission geometry on thin ice layers as well as measurements in diffuse reflection geometry using a Fourier-transform near-infrared spectrometer (FTNIR)[2]. Overlapping frequency range of the two instruments is the combinational mode ($\nu_{OH}+\nu_2$, $\nu_{OH}+2\nu_R$) at around 5000 cm^{-1}. The samples are prepared and measured independently at Stockholm University and University of Innsbruck, respectively. A comparison of eHDA is presented in Figure 2. Here we additionally compare eHDA as well as LDA (Figure S6B). We observe a slight shift comparing the spectra, this is the diffuse

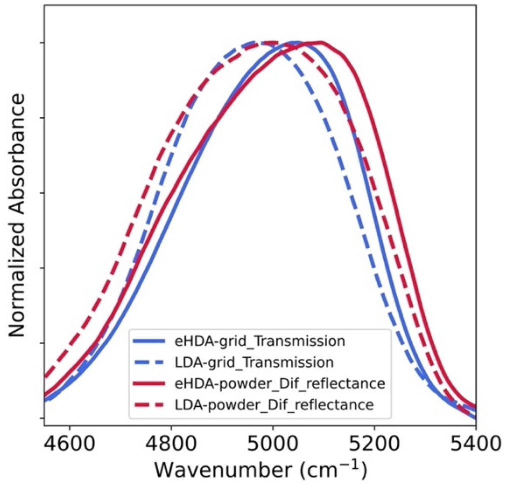

Figure S6B: Comparison of eHDA and LDA measured by FTIR in transmission geometry (ice made as a thin layer) as well as diffusive reflectance on powdered bulk samples. All spectra were measured at 80 K.

reflectance spectra has a larger FWHM than spectra obtained from transmission geometry measurements. The peak position for both, eHDA and LDA is shifted slightly towards higher wavenumbers than in transmission FTIR spectroscopy.

Coexistence of LDA and HDA:

In the main manuscript we report that coexistence of eHDA and LDA is observed at 120 K. We here investigate further the fraction of the two components at different temperatures. We observe appearance of low-density structure at 90 K* (10 %), 105 K* (15 %), and 115 K* (30 %) in OD-stretch mode (ν_{OD}) shown in Fig. S7. (*Measurements are taken at 80 K after heating to corresponding temperatures and annealing for 10min.) Similar slow appearance of LDA fraction at lower temperatures were observed from X-ray diffraction measurements in *Mariedahl et al.*[6] using powdered eHDA samples. We could not apply the same analysis to the combinational mode ($\nu_{OH}+\nu_2$, $\nu_{OH}+2\nu_R$), since the transition affects the combinational spectra differently, due to different influence of bending and rotational vibration modes. For instance, it is known, that the FWHM of LDA of the combinational mode is larger than FWHM of eHDA, which is opposite to the OD-stretch spectra.

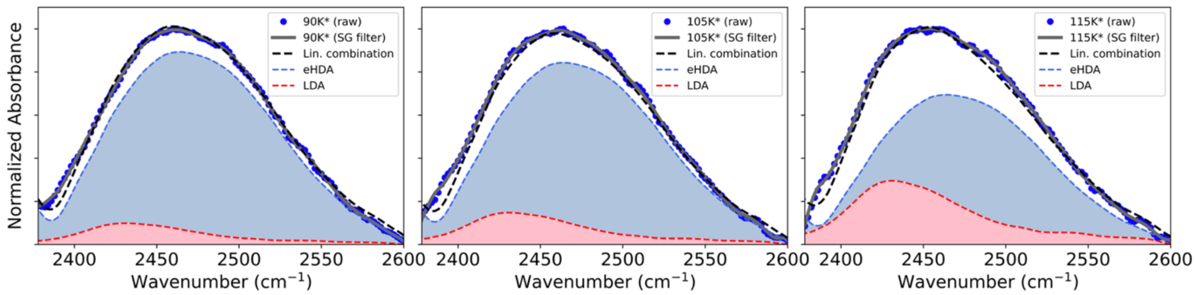

Figure S7. OD stretch mode at different temperatures decomposed in LDA and eHDA fraction.

References

(1) Tulk, C. A.; Klug, D. D.; Branderhorst, R.; Sharpe, P.; Ripmeester, J. A. Hydrogen Bonding in Glassy Liquid Water from Raman Spectroscopic Studies. *The Journal of Chemical Physics* **1998**, *109* (19), 8478–8484. https://doi.org/10.1063/1.477512.

(2) Tonauer, C. M.; Köck, E.-M.; Gasser, T. M.; Fuentes-Landete, V.; Henn, R.; Mayr, S.; Kirchler, C. G.; Huck, C. W.; Loerting, T. Near-Infrared Spectra of High-Density Crystalline H 2 O Ices II, IV, V, VI, IX, and XII. *The Journal of Physical Chemistry A* **2021**, *125* (4), 1062–1068. https://doi.org/10.1021/acs.jpca.0c09764.

(3) Bertie, J. E.; Labbé, H. J.; Whalley, E. Infrared Spectrum of Ice VI in the Range 4000–50 Cm−1. *The Journal of Chemical Physics* **1968**, *49* (5), 2141–2144. https://doi.org/10.1063/1.1670377.

(4) Bertie, J. E.; Whalley, E. Infrared Spectra of Ices II, III, and V in the Range 4000 to 350 Cm—1. *The Journal of Chemical Physics* **1964**, *40* (6), 1646–1659. https://doi.org/10.1063/1.1725374.

(5) Li, H.; Karina, A.; Ladd-Parada, M.; Späh, A.; Perakis, F.; Benmore, C.; Amann-Winkel, K. Long-Range Structures of Amorphous Solid Water. *The Journal of Physical Chemistry B* **2021**, *125* (48), 13320–13328. https://doi.org/10.1021/acs.jpcb.1c06899.

(6) Mariedahl, D.; Perakis, F.; Späh, A.; Pathak, H.; Kim, K. H.; Benmore, C.; Nilsson, A.; Amann-Winkel, K. X-Ray Studies of the Transformation from High- To Low-Density Amorphous Water. *Philosophical Transactions of the Royal Society A: Mathematical, Physical and Engineering Sciences* **2019**. https://doi.org/10.1098/rsta.2018.0164.
